# Lung ultrasound for the diagnosis and monitoring of community-acquired pneumonia in children: a prospective observational study

**DOI:** 10.3389/fped.2025.1702388

**Published:** 2025-12-10

**Authors:** M. Francavilla, L. Scarlato, A. Camporesi, A. Orlandi, C. Cafagno, C. L. Raguseo, V. Santoiemma, R. Russo, A. Sacco, A. M. Musolino, M. C. Supino, A. Clemente, L. Tagliaferri, R. Morello, V. Greco Miani, C. Bisceglia, G. Stellacci, D. Caselli, D. Buonsenso

**Affiliations:** 1 Department of Radiology, Giovanni XXIII Children Hospital, Azienda Ospedaliero Universitaria Consorziale Policlinico, Bari, Italy; 2 Pediatric Infectious Diseases Unit, Giovanni XXIII Children Hospital, Azienda Ospedaliero Universitaria Consorziale Policlinico, Bari, Italy; 3 Pediatric Anesthesia and Intensive Care, Vittore Buzzi Children’s Hospital, Milano, Italy; 4 Bambino Gesù Children’s Hospital IRCCS, Hospital University Pediatrics Clinical Area, Rome, Italy; 5 UOC Radioterapia Oncologica, Dipartimento Di Diagnostica Per Immagini, Radioterapia Oncologica Ed Ematologia, Fondazione Policlinico Universitario A. Gemelli, IRCCS, Rome, Italy; 6 Area Pediatrica, Dipartimento di Scienze Della Vita e Sanità Pubblica, Università Cattolica del Sacro Cuore, Rome, Italy; 7 Department of Woman and Child Health and Public Health, Fondazione Policlinico Universitario A. Gemelli IRCCS, Rome, Italy

**Keywords:** lung, ultrasound, pneumonia, pediatrics, diagnosis, monitoring

## Abstract

**Introduction:**

Community-acquired pneumonia remains a noteworthy concern in pediatrics due to the difficulty in identifying the underlying etiology and the risk of complications associated with high morbidity and mortality.

**Methods:**

This is a multicenter, prospective study that enrolled 315 children admitted to three different hospitals in Italy between March 2023 and June 2024. The ultrasound scans were performed according to the approach proposed by Soldati et al. in 2020 at admission (T0) and during hospitalization (T1).

**Results:**

Lung ultrasound proved to be a valuable tool for differentiating etiologies, with significantly higher scores observed in bacterial and viral pneumonias compared to atypical cases (respectively *p* < 0.001 and *p* = 0.018). Furthermore, elevated ultrasound scores were predictive of adverse outcomes, such as the need for respiratory support or admission to the intensive care unit (*p* < 0.05). Finally, our findings highlight the importance of timing in follow-up assessments: a 72-hour interval was sufficient to detect improvements in lung ultrasound findings in severe cases (*p* < 0.01), while it appeared unnecessary in mild ones (*p* = 0.139).

**Discussion:**

This study confirms the usefulness of lung ultrasound in monitoring children with community-acquired pneumonia and in the early identification of those at risk of clinical deterioration. Moreover, our findings offer useful guidance for optimizing follow-up timing, supporting personalized ultrasound approaches tailored to disease severity while reducing unnecessary examinations.

## Introduction

Community-acquired pneumonia (CAP) is a major public health concern in the pediatric population, as it is the leading cause of hospitalization and death in children younger than 5 years worldwide (1). Moreover, the etiological diagnosis of CAP remains a major challenge. It is usually based on a combination of clinical, laboratory, and radiological findings, although these are often insufficient (2, 3). Recent studies suggest that lung ultrasound (LUS) may play a crucial role in the diagnosis of pediatric community-acquired pneumonia (CAP). This emerging tool can support clinicians in differentiating between viral, bacterial, and atypical forms, thereby guiding appropriate therapy and consequently reducing unnecessary antibiotic use, hence antibiotic resistance (4, 5). LUS is also a valuable tool for monitoring the progression of CAP, representing a sensitive and specific alternative to chest x-ray, with the important advantage of being radiation-free (1, 6, 7).

Considering the significant burden of CAP in the pediatric population, the persistent challenges in its diagnosis, and the growing evidence supporting the role of lung ultrasound (LUS), this study aims to evaluate the application of LUS in the diagnosis and follow-up of CAP in a multicenter pediatric cohort. In particular, we investigate its role in predicting the evolution of pneumonia according to different etiologies and severity.

## Method

We performed a multicenter, prospective, observational study involving three Italian hospitals. Children aged 1 month to 17 years, admitted between March 2023 and June 2024 with a clinical diagnosis of CAP, were enrolled. Medical history, clinical information, laboratory, radiological and microbiological data were collected.

### Etiological classification

Patients were classified into four groups (bacterial pneumonia, viral pneumonia, atypical pneumonia, and pneumonia of unknown origin) by a panel of three infectious disease specialists who were blinded to discharge summaries. The panel reviewed the final dataset and assigned an etiological diagnosis based on predefined clinical, radiological, and microbiological criteria, detailed as follows.

- Bacterial pneumonia
  ○ *Confirmed bacterial pneumonia* was diagnosed when a bacterial pathogen was identified by culture or PCR from clinically relevant samples (e.g., bronchoalveolar lavage, pleural fluid, or blood).
  ○ *Probable bacterial pneumonia* was diagnosed in the absence of microbiological confirmation, when at least two of the following criteria were met: lobar consolidation or significant pleural effusion on chest x-ray; leukocytosis (>15 × 10^9^ /L); elevated inflammatory markers (C-reactive protein >40 mg/L or procalcitonin >2 ng/mL).
  ○ This classification was maintained even when viral or atypical pathogens were detected in nasopharyngeal swabs, to account for potential co-infections.
- Viral pneumonia
  ○ *Confirmed viral pneumonia* was diagnosed when a viral pathogen was identified in the nasopharyngeal swab only after bacterial co-infection was carefully ruled out.
  ○ *Probable viral pneumonia* was diagnosed in the absence of viral microbiological confirmation and bacterial criteria, when at least two of the following criteria were met: upper respiratory tract symptoms (e.g., rhinorrhea, congestion, non-purulent conjunctivitis); diffuse and bilateral auscultatory findings; wheezing; age ≤2 years.
- Atypical pneumonia:
  ○ *Confirmed atypical pneumonia* was diagnosed when an atypical pathogen (Mycoplasma pneumoniae or Chlamydia pneumoniae) was identified by culture or PCR from pharyngeal swabs or by serological testing, in the absence of bacterial co-infection. If a viral co-infection was present, the patient was still included in this group.
  ○ *Probable atypical pneumonia* was diagnosed when atypical pathogens were not detected and bacterial criteria were not met, but at least two of the following criteria were present: constitutional symptoms (e.g., malaise, myalgia, headache, photophobia, sore throat, hoarseness, conjunctivitis, rash); a non-productive cough lasting more than 7 days and gradually worsening; lack of clinical improvement after 48 h of aminopenicillin therapy; age ≥5 years.
- Pneumonia of unknown origin:
  ○ Patients who did not meet the criteria for any of the above categories were classified as having pneumonia of unknown origin. This group also included patients with mild symptoms who could not be clearly classified as “probable atypical” or “probable viral.”

Amoxicillin-clavulanate was the first-choice oral antibiotic therapy, in line with the guidelines for the treatment of CAP. Macrolides, such as Clarithromycin, were used in cases of suspected atypical pneumonia. In more clinically severe forms of pneumonia, or when oral therapy was not tolerable, intravenous Ceftriaxone was administered (8, 9).

### Lung ultrasound

For each patient, an initial LUS examination was performed within six hours of the first clinical assessment (T0). A follow-up ultrasound examination was conducted after an interval of 2–4 days (T1). Lung ultrasound was performed by either a radiologist or a pediatrician. All clinicians involved in the study had previously completed formal theoretical–practical training courses in lung ultrasound and had at least three years of clinical experience in this field. The scans were performed following the approach proposed by Soldati et al. in 2020, in which a score was obtained by summing the scores of the different findings (consolidations, bronchograms, vertical artifacts and pleural effusion) detected in 14 lung fields (10).

Lesions were defined as follows:

- Consolidation: area in which lung tissue is de-aerated, with density similar to parenchymal tissues.
- Pleural effusion: hypo- or anechogenic structure delineated by the chest wall and the diaphragm.
- B-lines: laser-like, vertical hyperechogenic artefacts originating from the pleural line and extending to the edge of the screen, synchronized with the pleural line.

The total LUS score is reported in detail below (11, 12).

- Short vertical artifacts were excluded from scoring.
- B lines were scored as follows:
  ○ Isolated: 1 point
  ○ Confluent: 2 points
- White lung pattern: 3 points, regardless of location or extent.
- Consolidations were assessed based on:
  ○ Presence: 4 points
  ○ Size:
    ▪ <1 cm: 0 points
    ▪ 1–3 cm: 1 point
    ▪ 3 cm: 2 points
  ○ Distribution:
    ▪ Bilateral: 1 point
    ▪ Unilateral: 2 points
  ○ The number of consolidations did not affect the score.
- Air bronchograms (if present) were scored based on type and depth:
  ○ Static: 1 point
  ○ Dynamic: 1 point
  ○ Superficial (<2 cm from the pleura): 1 point
  ○ Deep (>2 cm): 2 points
- Fluid bronchograms:
  ○ Absent: 0 points
  ○ Present: 1 point
- Pleural effusions:
  ○ None: 0 points
  ○ Small: 0.5 points
  ○ Moderate/Large: 1 point
  ○ Type:
    ▪ Simple: 1 point
    ▪ Complex: 2 points

### Statistical analysis

Categorical variables were presented as counts and percentages, while continuous variables were expressed as mean (SD) or medians (IQR) according to their distribution. Comparisons of symptom prevalence between baseline (T0) and follow-up (T1) were performed using McNemar's test for paired categorical data. Longitudinal changes in lung ultrasound (LUS) scores were analyzed using mixed linear regression models, accounting for within-subject correlation by including random effects. All tests were two-sided, and statistical significance was set at *p* < 0.05. Analyses were conducted using STATA v.19.5 SE (StataCorp, US).

## Results

### Patient characteristics and treatment

A total of 315 children were included in the study: 90 with confirmed/probable viral pneumonia, 72 with confirmed/probable bacterial pneumonia, 11 with confirmed/probable atypical pneumonia and 142 with pneumonia of unknown origin. A total of 179 patients received oral antibiotics (including bacterial pneumonias and nearly half of those with viral or undefined pneumonia), while 90 patients received intravenous antibiotics (including bacterial pneumonias, 12 cases of viral pneumonia and 69 cases of pneumonia of unknown origin). The first-line oral antibiotic was Amoxicillin-clavulanate (64 patients, 57%), followed by Clarithromycin (19 patients, 17%). In the patients receiving intravenous antibiotics, Ceftriaxone was the most common (52 patients, 58%), followed by Amoxicillin/Clavulanate (24 patients, 27%).

### LUS score at admission (T0)

LUS score was higher in bacterial and viral pneumonia (respectively mean 35.44, SD 20.16 and mean 29.34, SD 15.27) compared with atypical pneumonia (mean 16.90, SD 12.34): there was a linear relationship between LUS score at admission and the etiology of pneumonia (confirmed/probable bacterial pneumonia coefficient 13.38, 95% CI: 7.27–19.49, *p* < 0.001; confirmed/probable viral pneumonia coefficient 7.29, 95% CI: 1.27–13.30, *p* = 0.018).

Moreover, patients who received Amoxicillin-clavulanate presented a higher LUS score (31.87, SD 17.64) compared with patients treated with macrolides (Clarithromycin: 21.41, SD 14.92; Azithromycin 6.28, SD 5.31), probably reflecting the different etiologies behind the different therapeutic choices.

LUS score at admission was also correlated with the level of care during hospitalization, such as the need for ward or PICU admission, oxygen therapy, CPAP, or invasive ventilation: every point in LUS score increased the relative risk of ward admission by 5% (RRR 1.05, 95% CI: 1.03–1.07, *p* < 0.001) and of pediatric intensive care unit (PICU) admission by 8% (RRR 1.08, 95% CI: 1.05–1.11, *p* < 0.05), the need for low flow oxygen by 2% (RRR 1.02, 95% CI: 1.01–1.04, *p* < 0.001), the need for CPAP by 5% (RRR 1.05, 95% CI: 1.02–1.08, *p* < 0.001), and the need for invasive ventilation by 6% (RRR 1.06, 95% CI: 1.03–1.10 *p* < 0.05).

Patients were categorized according to their LUS score at admission into “severe” (LUS score greater than or equal to 30) and “not severe” (LUS score less than 30). At T0, 53/90 viral pneumonias were severe, as well as 42/72 bacterial and 2/11 atypical (respectively 59% of viral, 58% of bacterial and 18% of atypical). Being “severe”, regardless of the etiology, had a relative risk of 3.08 for starting low flow oxygen (95% CI: 1.54–6.15, *p* < 0.001) and of 6.5 for invasive ventilation (95% CI: 1.35–31.10, *p* = 0.019).

### Follow-up assessment (T1)

A total of 212 patients were reevaluated at T1. In 103 patients, rapid clinical improvement led to discharge before 48 h, therefore T1 reevaluation could not be performed. T1 assessment was performed at 3.17 (SD 1.76) days after T0 (median 3, IQR 2–4). LUS score at T1 was still associated with need for CPAP (RRR 1.04, 95% CI: 1.06–1.08, *p* < 0.05) and invasive ventilation (RRR 1.07, 95% CI: 1.03–1.11, *p* < 0.05).

Comparing observations at T0 and T1 revealed a significant reduction in all clinical manifestations (*p* < 0.05), as shown in Table 1.

**Table 1:** Clinical manifestations at T0 and T1.

Clinical manifestations	T0	T1	*P* value
Fever	207/316 (65.5%)	11/211 (5.2%)	<0.001
Cough	300/314 (95.5%)	170/211 (80.5%)	<0.001
Dyspnea	126/314 (40.1%)	44/212 (20.7%)	<0.001
Wheezing	75/312 (24%)	29/212 (13.7%)	0.0016
Crackles/rales	271/318 (85%)	152/215 (70.7%)	<0.001
Reduced ventilation	93/313 (29.7%)	50/213 (23.5%)	<0.001

Similarly, the LUS score decreased between T0 and T1. The mean LUS score at T0 was 26.7 (±21.02; median 23, IQR 10–40), while at T1 it was 20.64 (±20.43; median 17, IQR 6–29). This difference was statistically significant (*p* = 0.0023).

### Mixed linear model analysis

We performed a mixed linear model analysis on the total LUS score (the sum of the scores obtained at T0 and T1), including time and etiology as covariates. In the mixed linear model, the mean LUS score was significantly lower at T1 compared with T0 across the whole cohort (coefficient −7.41, 95% CI: −11.32 to −3.50, *p* < 0.001). Patients with confirmed or probable viral pneumonia (coefficient 7.29, 95% CI: 1.44–13.14, *p* = 0.015) and confirmed or probable bacterial pneumonia (coefficient 13.38, 95% CI: 7.44–19.32, *p* < 0.001) had higher baseline LUS scores than the reference group (undefined pneumonia), whereas the atypical pneumonia group did not differ significantly (coefficient: −5.15, 95%, CI: 17.36–7.05, *p* = 0.408). Interaction terms between time and pneumonia etiology were not statistically significant, indicating that the reduction in LUS scores over time was comparable across diagnostic groups.

**Figure 1:**
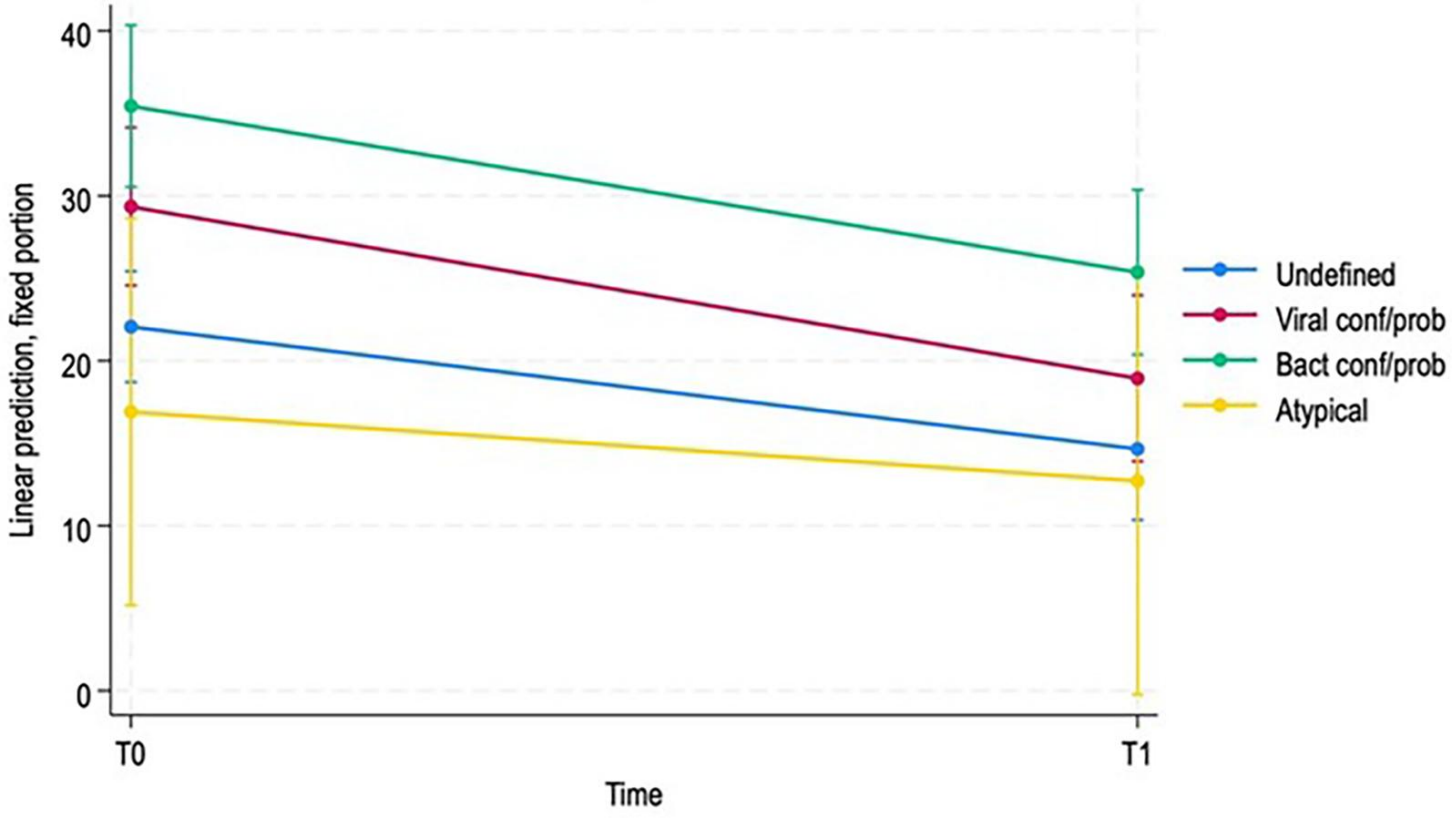
Predicted evolution of LUS score over time depending on different etiologies.

Figure 1 shows the expected decrease in LUS score over time, depending on the underlying etiology.

Finally, the same model was repeated including ultrasound severity as a covariate. Patients classified as severe had significantly higher baseline LUS scores compared to non-severe cases (coefficient 42.18, 95% CI: 36.75–47.615, *p* < 0.001). Among severe patients, the reduction of the score at T1 was much larger (coefficient −15.65, 95% CI: −22.68 to −8.62, *p* < 0.001), whereas the change over time was not significant in the non-severe group (coefficient −3.26, 95% CI: −7.59 to 1.07, *p* = 0.139). Viral and bacterial pneumonias were associated with higher baseline scores (viral coefficient 7.71, 95% CI: 2.39–13.03, *p* = 0.005; bacterial coefficient 7.97, 95% CI: 2.22–13.723, *p* = 0.007, respectively). Interactions between severity and pneumonia etiology showed that these diagnostic effects were attenuated in severe patients. The three-way interactions between pneumonia etiology, time, and severity were not statistically significant, indicating that the magnitude of improvement from T0 to T1 was consistent across diagnostic categories.

**Figure 2:**
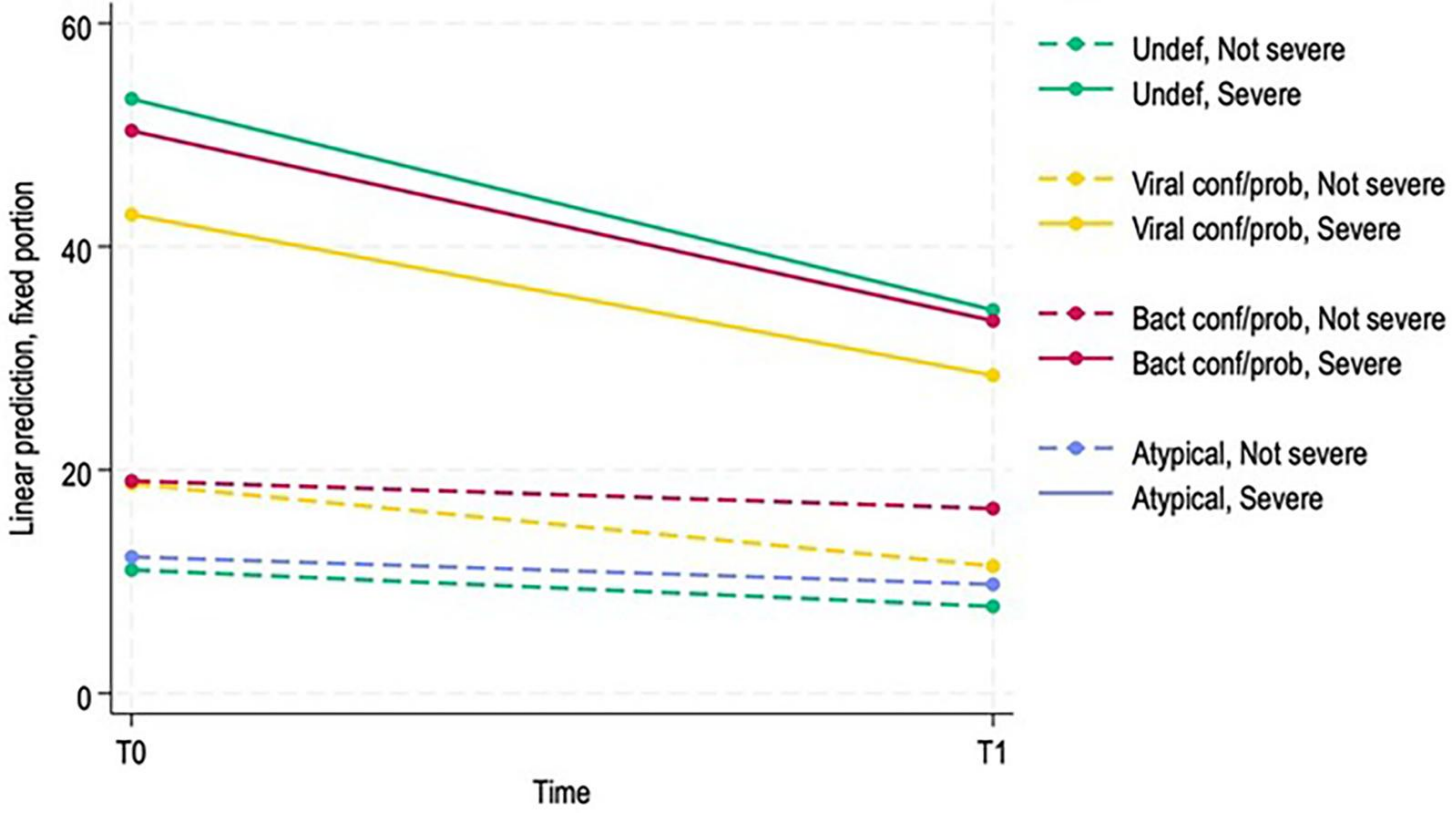
Predicted evolution of LUS score over time depending on different etiologies and severity.

Figure 2 shows the expected evolution of LUS score over time, according to etiology and severity. The atypical severe line is not shown in the figure due to the small number of patients in this subgroup.

## Discussion

Our study shows the relevant role of LUS in differentiating the etiologies of CAP, revealing the highest ultrasound score in bacterial pneumonia, followed by viral pneumonia and lastly atypical pneumonia, in line with recent literature (4, 5). Even more importantly, LUS scores at admission and at follow-up were able to predict the development of complications such as the need for respiratory support or admission to the PICU. A limited number of previous studies investigated the evolution of LUS findings over time, suggesting the ability of sonography features to predict the worsening of pneumonia more than clinical manifestations and laboratory data (13), and the possible use of LUS in early detection of complications like ventilator-associated pneumonia (14) or necrotizing pneumonia (15). Additionally, Shereen A. Mohamed et al. demonstrated that lung ultrasound (LUS) has high sensitivity (88.1%) and specificity in diagnosing pneumonia, outperforming chest x-ray (50%) and showing comparable accuracy to chest CT (95.2%) (7). Moreover, LUS can guide antimicrobial therapy and thus contribute to better clinical outcomes. In their study, 26% of patients had their antimicrobial regimen modified based on imaging results, specifically LUS and CT, without waiting for endotracheal aspirate results (7). Our study confirmed these findings, indicating that severe ultrasound patterns and a consequent high LUS score warrant careful clinical monitoring.

Patients enrolled in the study were reevaluated after a mean of 3 days from the first observation. This period was sufficient to notice a significant clinical improvement, and a parallel reduction of LUS score. Similar results were reported by Isac et al, considering a second examination performed after 72–120 h from admission (16). However, in our study, LUS score evolution was significantly correlated with time, suggesting that a longer time interval between the ultrasound examinations could better detect the evolution of pneumonia, regardless of etiology. Nir et al. examined the use of LUS as a monitoring tool in children with CAP, reporting that LUS proved to be useful in identifying the resolution of pneumonia when performed 10–14 days after diagnosis (17). Moreover, when we investigated the effect of severity on LUS score evolution, we found that over a short time interval, improvement was significant only for severe forms of pneumonia, while non-severe patients tended to maintain a stable ultrasound score. Our findings suggest that, in case of low baseline LUS score, close monitoring that includes a second LUS examination after only 48–72 h is neither necessary nor useful, unless complications are suspected.

### Strengths and limitations

This study has some limitations. Microbiological stratification may be imprecise, as it often relied on nasopharyngeal samples rather than invasive procedures such as bronchoalveolar lavage. In many cases, microbiological tests showed negative results, leaving the etiological diagnosis undetermined. Moreover, the small number of patients in each subgroup, particularly those with confirmed or probable atypical pneumonia, limited the statistical power to detect significant differences.

Although some degree of inter-operator variability cannot be entirely excluded, all examinations were performed following a standardized protocol. The methodology, adapted from Soldati et al. (10), included a predefined topographic scanning scheme, detailed characterization of each pulmonary finding, and the application of a uniform grading system. Adherence to this structured and reproducible approach minimizes variability both within and across participating centers.

Despite these limitations, to our knowledge, this is the first study to assess the role of LUS in monitoring pneumonia across well-defined etiological subgroups in a pediatric population. These findings highlight the value of lung ultrasound not only in diagnosis, but also as a supportive tool for predicting clinical outcomes and guiding patient management, alongside clinical and laboratory assessments.

### Implications for policy, practice, and research

This research demonstrates the importance of LUS in monitoring children with CAP and predicting possible clinical deterioration. Furthermore, our results provide valuable insights for the optimization of the timing of CAP follow-up, allowing for individualized ultrasound assessment strategies based on pneumonia severity and minimizing unnecessary procedures. Further studies are needed to validate and expand our results in larger pediatric populations.

## Data Availability

The raw data supporting the conclusions of this article will be made available by the authors, without undue reservation.
